# The Roles and Evolutionary Patterns of Intronless Genes in Deuterostomes

**DOI:** 10.1155/2011/680673

**Published:** 2011-08-11

**Authors:** Ming Zou, Baocheng Guo, Shunping He

**Affiliations:** ^1^The key Laboratory of Aquatic Biodiversity and Conservation of Chinese Academy of Sciences, Institute of Hydrobiology, Chinese Academy of Sciences, Wuhan 430072, China; ^2^Institute of Hydrobiology, Graduate University of the Chinese Academy of Sciences, Beijing 100039, China; ^3^Institute of Evolutionary Biology and Environmental Studies, University of Zurich, 8057 Zurich, Switzerland; ^4^The Swiss Institute of Bioinformatics, Quartier Sorge-Batiment Genopode, 1015 Lausanne, Switzerland

## Abstract

Genes without introns are a characteristic feature of prokaryotes, but there are still a number of intronless genes in eukaryotes. To study these eukaryotic genes that have prokaryotic architecture could help to understand the evolutionary patterns of related genes and genomes. Our analyses revealed a number of intronless genes that reside in 6 deuterostomes (sea urchin, sea squirt, zebrafish, chicken, platypus, and human). We also determined the conservation for each intronless gene in archaea, bacteria, fungi, plants, metazoans, and other eukaryotes. Proportions of intronless genes that are inherited from the common ancestor of archaea, bacteria, and eukaryotes in these species were consistent with their phylogenetic positions, with more proportions of ancient intronless genes residing in more primitive species. In these species, intronless genes belong to different cellular roles and gene ontology (GO) categories, and some of these functions are very basic. Part of intronless genes is derived from other intronless genes or multiexon genes in each species. In conclusion, we showed that a varying number and proportion of intronless genes reside in these 6 deuterostomes, and some of them function importantly. These genes are good candidates for subsequent functional and evolutionary analyses specifically.

## 1. Introduction

Most eukaryotic genes are interrupted by one or more noncoding sequences called introns, and intronless genes are a characteristic feature of prokaryotes. However, researches on intronless genes in eukaryotes have been reported over the past few decades [1–4]. Many human genes, like G protein-coupled receptor genes, are intronless [5] and the human genome report identified 901 predicted intronless genes [6]. Recently, Tay et al. found that many single-copy primate-specific human transcriptional units are single exon [7]. Moreover, Yang et al. found that species-specific genes in *Arabidopsis*, *Oryza,* and *Populus* are enriched with intronless genes [8]. A retrogene, which is formed by homologous recombination between the genomic copy of a gene and an cDNA [9], is also considered to be intronless, and it has been reported that many retrogenes exist in eukaryotic genomes [10–12]. Intronless genes in eukaryotes, because of their prokaryotic architecture, provide interesting datasets for comparative genomics and evolutionary studies. Studying these genes can help to understand the evolutionary patterns of related genes and genomes. As a result, systematical researches on intronless genes in many species from mammals to plants have been reported [13–18]. Several databases of these single exon genes, such as SEGE [19] and Genome SEGE [20], have been set up and are of important use for evolutionary and functional studies. However, former evolutionary researches on intronless genes have usually been limited to 1 to 2 species and studies within a phylogenetic framework are rare. With the development of sequencing technology, more and more complete genomes have been sequenced and annotated, which makes comprehensive comparative analysis on intronless genes possible. The present study was designed to identify and analyse intronless genes in 6 deuterostomes, sea urchin (*Strongylocentrotus purpuratus*), sea squirt (*Ciona intestinalis*), zebrafish (*Danio rerio*), chicken (*Gallus gallus*), platypus (*Ornithorhynchus anatinus*), and human (*Homo sapiens*), which were selected because of their pivotal phylogenetic positions. We compared the functions and conservation of these genes between and within species in an attempt to gain some evolutionary meaningful insights.

## 2. Materials and Methods

### 2.1. Data Source of Intronless Genes

The annotated genomes (GenBank Flat File Format) of sea urchin, sea squirt, zebrafish, chicken, platypus, and human were downloaded from the NCBI ftp server (ftp://ftp.ncbi.nih.gov/genomes/, 10 Jun 2009). Using a customized Perl script, we extracted protein sequences for all the intron and intronless genes from each annotated genome. During our processing, a gene was classified as intron-containing if the “CDS” line in the FEATURES contains a “join”; otherwise, it was classified as an intronless gene. Proteins that encoded by mitochondrial genomes were removed. To avoid any ambiguity, proteins encoded by genes which have the symbol “<” or “>” in their annotation (“<” indicates partial on the 5′ end and “>” indicates partial on the 3′ end) were also discarded.

### 2.2. Functional Assignment and Category

ProtFun is an online procedure designed to produce *ab initio *predictions of protein functions from sequences and combines 14 different sequence-based functional prediction methods. ProtFun queries a large number of other feature prediction servers to obtain information on various posttranslation and localisation aspects of the protein to predict protein function, rather than relying on sequence similarity compared with other protein function prediction procedures [21, 22]. Therefore, functional assignments of intronless genes in our study were done with the webserver ProtFun (http://www.cbs.dtu.dk/services/ProtFun/) and sequences were clustered according to their cellular roles and gene ontology (GO) categories.

### 2.3. Distribution, Conservation, and Paralogue Identification of Intronless Genes

Genes (both intronless and intron-containing genes) in archaea, bacteria, fungi, plants, metazoans, and other eukaryotes homologous with our intronless genes (BLAST score more than 100), were determined on the basis of sequence similarity using BLink (BLAST Link), which is a tool that displays the precomputed results of BLAST searches that have been completed for every protein sequence in the Entrez proteins data domain [24] and is available at NCBI.

CD-HIT is a program for clustering the entries in a large protein database according to sequence identity (with a high threshold of identity). CD-HIT can remove redundant sequences and generate a database of only the representatives [25]. To determine the conservative intronless genes among the 6 deuterostome species in this study, we clustered all of our intronless genes using CD-HIT. In order to determine the relationships among these intronless genes, we clustered them and identified nonredundant intronless genes in each genome. We also clustered intron-containing genes to produce nonredundant multiexon genes in each genome. We clustered these nonredundant intronless genes with nonredundant multiexon genes in the same genome and produced a list of corresponding intronless and intron-containing genes to determine the relationships between intronless and intron-containing genes. All these data handling were done with CD-HIT.

## 3. Results and Discussion

### 3.1. Intronless Genes in Deuterostomes

Sea urchin, sea squirt, zebrafish, chicken, platypus, and human were selected to represent the major groups of deuterostomes and the intronless genes in their genomes were identified. Gi number and protein sequence for each intronless gene in each species were obtained from processing their annotated genomes. As a result, there are abundant intronless genes in each of the 6 deuterostome genomes. The numbers of intronless genes in each species is given in Table 1 and details are given in supplementary material online at doi:10.1155/2011/680673. Among the selected species, human has the maximum number of intronless genes (6229) and platypus has the least (930). We can see the maximum one is nearly seven times the number of the least one. However, the difference among numbers of intronless genes in sea urchin (2482), zebrafish (2169), chicken (1659), and sea squirt (1448) is not significant and these numbers should increase and be more accurate when their well-annotated genomes are available. Since a few previous studies reported a bit lower numbers of the number of intronless gene [16, 20], we compared protein numbers (encoded by intron and intronless genes) from Ensemble (Table 1) with ours, and found the former was always larger. Compared to their numbers, proportions that intronless genes are accounting for total genes do not differ significantly, and the maximum one is about twice the number of the least one (Table 1). In fact, former researches reported that 11109, 5846, and 5085 intronless genes reside in rice, *Arabidopsis* and mouse genomes, accounting for 19.9%, 21.7%, and 18.9% genes correspondingly [13]. Given that the total gene numbers and annotation qualities between species are different, these data may indicate that although the number of intronless genes varied significantly between species, the proportions that they account for total genes are nearly constant. However, the number and percentages of intronless genes do not correlate with their genome sizes (*P* > 0.6, |*r* | < 0.3, Spearman's test). The human genome has the largest number of intronless genes, which might be due to the following reasons. Firstly, human has the most complete expression data, which could result in more annotated genes compared with other species during the genome annotation process. Secondly, the human genome has many more retrogenes compared with other species [26, 27]. Thirdly, duplications of intronless genes are common in the human genome (see later). Plenty of intronless genes exist in the 6 deuterostomes indicating they may play important roles during deuterostome evolution. Earlier, Jain et al. found that intronless genes have a strong bias towards encoding shorter proteins [13]. Here we testified that the average length of intronless genes is significantly shorter than multiexon genes in all the selected species (*P* < 0.001, Mann-Whitney Test). The average length for intronless genes in sea urchin, sea squirt, zebrafish, chicken, platypus, and human is 341.75 bp, 389.34 bp, 378.78 bp, 259.53 bp, 294.43 bp, 241.26 bp, and for intron genes is 530.59 bp, 540.58 bp, 553.04 bp, 541.31 bp, 528.11 bp, and 503.31 bp, respectively.

**Figure 1:**
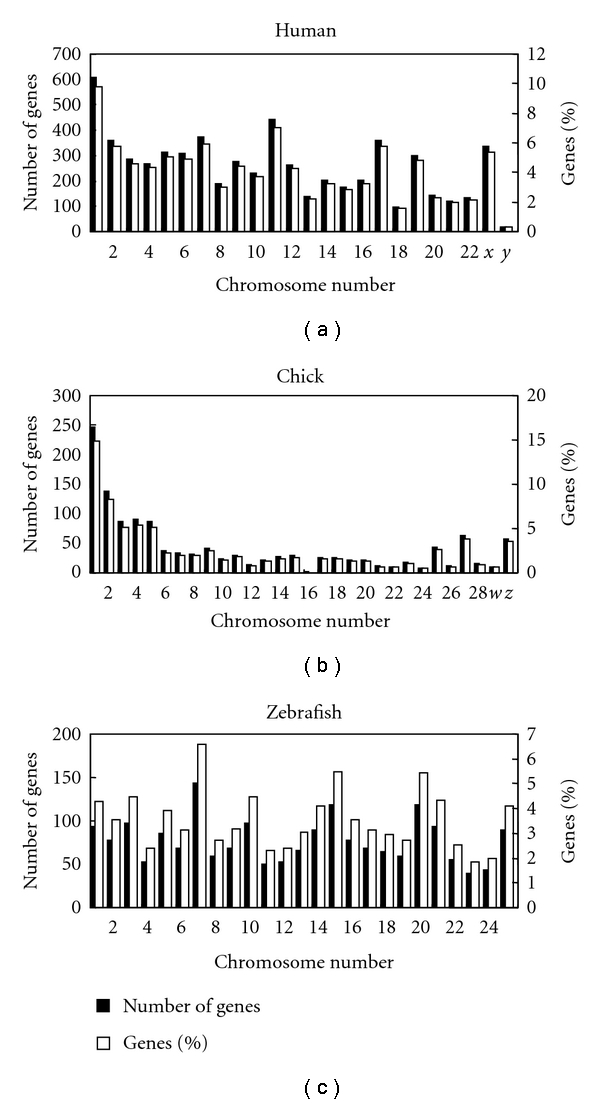
The numbers of intronless genes on each chromosome in human, chicken, and zebrafish. Both numbers and percentages are shown.

Among the selected species, chromosomes were well assembled in human, chicken, and zebrafish. To study the distribution of intronless genes in each selected genome, we counted the numbers of intronless genes on each of their chromosomes (Figure 1). Spearman's test showed that the number of intronless genes is significantly correlated with the length of their chromosomes in human (*P* < 0.001, *r* = 0.721) and chicken (*P* < 0.001, *r* = 0.712). The correlation may be also significant in zebrafish (*P* = 0.119, *r* = 0.320) given that nonparametric tests have less “power” to detect a significant difference. Therefore, we proved that the distributions of intronless genes in human and chicken (and maybe in zebrafish) are stochastic, just like previous studies in mouse, rice, and *Arabidopsis * [13, 18]. However, several clusters of intronless genes exist in certain chromosomes and some of these clusters have been reported. For example, the olfactory receptor gene clusters on human chromosome 17 and odorant receptor genes in the zebrafish genome [28, 29].

### 3.2. Functional Assignment of Intronless Genes

**Figure 2:**
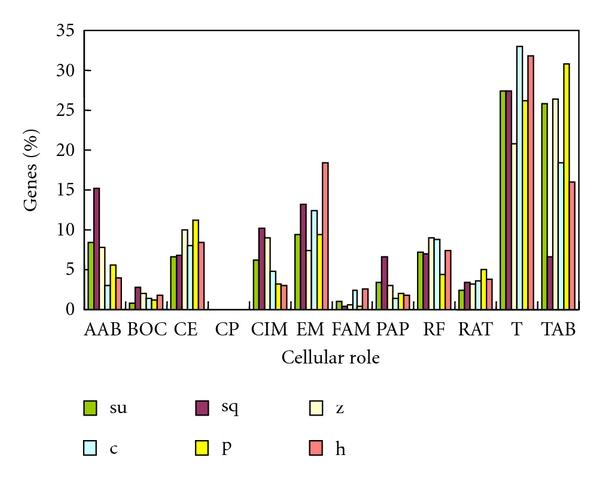
Distribution of intronless genes among different cellular roles in each species. Su: sea urchin; sq, sea squirt; z: zebrafish; c: chicken; p: platypus; h: human. AAB: amino acid biosynthesis; BOC: biosynthesis of cofactors; CE: cell envelope; CP: Cellular processes; CIM: Central intermediary metabolism; EM: energy metabolism; FAM: fatty acid metabolism; PAP: purines and pyrimidines; RF: regulatory functions; RAT: replication and transcription; T: translation; TAB: transport and binding.

**Figure 3:**
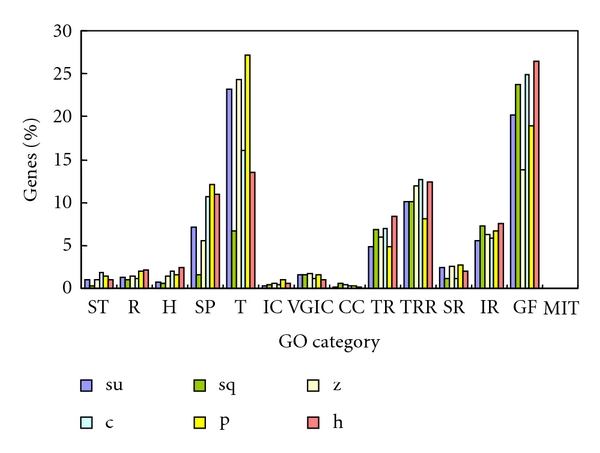
Distribution of intronless genes among different kinds of gene ontology (GO) categories in each species. su: sea urchin; sq: sea squirt; z: zebrafish; c: chicken; p: platypus; h: human. ST: signal transducer; R: receptor; H: hormone; SP: structural protein; T: transporter; IC: ion channel; VGIC: voltage-gated ion channel; CC: cation channel; TR: transcription; TRR: transcription regulation; SR: stress response; IR: immune response; GF: growth factor; MIT: metal ions transport.

It has been shown that the distribution of intronless human genes across molecular function categories is nonrandom [17]. In order to study the molecular function categories of intronless genes in the 6 selected species, their cellular roles and GO categories were predicted using ProtFun (available via webserver http://www.cbs.dtu.dk/services/ProtFun/).  Figure 2 shows the distribution of intronless genes among each cellular role in 6 species. As in plants, intronless genes that functionally belong to translation and energy metabolism are the commonest in most species, followed by the cell envelope and amino acid biosynthesis [13]. Furthermore, in these 6 deuterostomes, transport and binding, followed by regulatory functions and central intermediary metabolism, are also well represented compared with other function categories. The percentage of intronless genes with the same cellular role among the total intronless genes varies significantly between species (Figure 2). For example, 7% of intronless genes are transported and binding in sea squirt is significantly fewer than in other species. The number of cellular roles, such as amino acid biosynthesis and central intermediary metabolism, are quite similar in sea urchin, sea squirt, zebrafish, and human. However, this is not the case for chicken or platypus. GO categories can be assigned to more than 70% of intronless genes except in sea squirt (which is more than 60%), and the distribution of genes according to each GO category is shown in Figure 3. As in plants, proteins associated with the GO category growth factor, transcription regulation, transport, immune response and structural proteins are overrepresented in these species [13]. Furthermore, proteins associated with the GO category transcription, which might be different between plants and animals, are well represented in deuterostomes. The percentage of total intronless genes that proteins with a certain GO category, such as growth factor and transporter proteins, varied significantly among these species. According to their cellular roles and GO categories, the functional category distribution of intronless genes in each selected genome is very similar to those reported for rice and *Arabidopsis* [13]. This result might indicate that biological mechanisms related to intronless genes are common in the biological kingdom. On the basis of earlier work and this analysis, we concluded that most plant and deuterostome intronless genes have the same characteristics, but deuterostomes still have some lineage-specific and species-specific functional intronless genes.

### 3.3. Taxonomic Distribution

**Figure 4:**
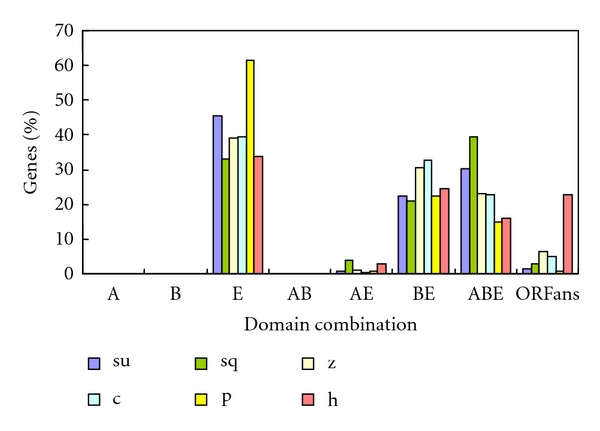
Distribution of intronless genes specific to different taxonomic group combinations for each species. su: sea urchin; sq: sea squirt; z: zebrafish; c: chicken; p: platypus; h: human. A: archaea; B: bacteria; E: eukaryote; AB: archaea and bacteria; AE: archaea and eukaryote; BE: bacteria and eukaryote; ABE: archaea, bacteria and eukaryote; ORFans: homologs not found in other organisms.

To study the evolutionary patterns of intronless genes in major taxonomic groups, we used BLink, a tool that displays the precomputed results of BLAST searches for every protein sequence from the entrez proteins data domain [24], to determine the evolutionarily conserved proteins among different taxonomic groups (archaea, bacteria, fungi, metazoans, plants, and other eukaryotes). The results of intronless gene clustering on the basis of homology with each taxonomic group are given in Table 2, and this will change as more genome sequences become available. We divided these genes into 7 types of combination according to their conservation among archaea (A), bacteria (B), and eukaryote (E) and the distributions are shown in Figure 4. Majority of intronless genes in each species that have homologues only in eukaryotes (E) suggested that most intronless genes emerged after the eukaryotes diverged from prokaryotes. Another important category of intronless genes is ABE, in which intronless genes are conserved in all major biological kingdoms, and these genes are considered to be functionally important and evolved slowly [30]. Intronless genes belonging to ABE account for 39% of the total intronless genes in sea squirt and 30% in sea urchin, which together form the first class. The second class contains zebrafish (23%), chicken (22%), and the third class includes human (16%) and platypus (14%). Given their phylogenetic positions, the first class is more primitive than the second class, which is more primitive than the third class. These data show that higher percentage of intronless genes in primitive species are inherited from the common ancestor of archaea, bacteria, and eukaryotes than in higher species. More than 20% of intronless genes are conserved in bacteria and eukaryotes (BE) in each species, but less than 5% are conserved in archaea and eukaryotes (AE). This could be because archaea have lost more homologues with eukaryotes than bacteria, or because bacteria have obtained more. Moreover, the percentage of genes conserved in bacteria and eukaryotes that account for total intronless genes is significantly higher in zebrafish and chicken than that in other species, suggesting that these 2 species have a greater percentage of intronless genes inherited from the common ancestor of bacteria and eukaryotes. No gene is conserved in archaea or/and bacteria except one human gene in bacteria and this might be an example of lateral gene transfer (LGT) from bacteria to human. 

**Figure 5:**
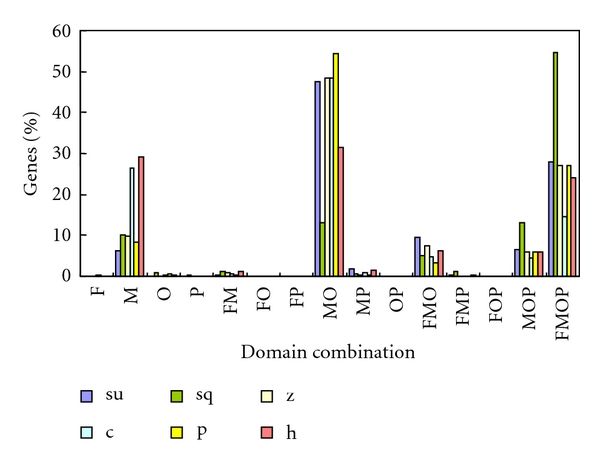
Distribution of eukaryote-specific intronless genes specific to different eukaryotic taxonomic group combinations for each species. su: sea urchin; sq: sea squirt; z: zebrafish; c: chicken; p: platypus; h: human. F: fungi; M: metazoans; O: other eukaryotes; P: plants; FM: fungi and metazoans; FO: fungi and other eukaryotes; FP: fungi and plants; MO: metazoans and other eukaryotes; MP: metazoans and plants; OP: other eukaryotes and plants; FMO: fungi, metazoans and other eukaryotes; FMP: fungi, metazoans and plants; FOP: fungi, other eukaryotes and plants; MOP: metazoan, other eukaryotes and plants FMOP: fungi, metazoans, other eukaryotes and plants.

More than 30% of intronless genes are eukaryote specific in all these species, especially in platypus (61.6%). To investigate their distributions in eukaryotic groups, we divided these proteins according to their homogeneity in fungi (F), metazoans (M), other eukaryotes (O), and plants (P) and formed 15 types of combination (Figure 5). Generally, majority of genes have homologues in the combination MO (metazoans and other eukaryotes) in each species except in sea squirt, in which only 13% of eukaryote-specific intronless genes are of this kind. Less than 10% of genes are metazoan-specific (M) in many species, but in chicken and human there are 26% and 29%, respectively. Genes conserved in fungi, metazoans, other eukaryotes, and plants (FMOP), including histones and ribosomal proteins, were thought to be very conservative because they are essential for the survival of all eukaryotes [30]. The number of FMOP genes is very similar in sea urchin (314), sea squirt (263) and zebrafish (228) but the percentage of total eukaryote-specific intronless genes is much greater in sea squirt (54.7%) than that in sea urchin (27.8%) and zebrafish (26.9%). Except those cases mentioned above, very few genes are conserved in other taxonomic combinations. Moreover, some genes in some species have homologues in fungi, plants or other eukaryotes but not in metazoans. These genes might be examples of lateral gene transfer (LGT) between eukaryotes, which has been demonstrated recently [31, 32]. Since fungi and plants diverged from metazoan ahead of other eukaryotes, the distribution pattern of eukaryote-specific intronless genes in these species can be explained by that much more homologs have been lost in fungi and plants plus lots of others have been obtained after their divergence. However, lots of essential genes (FMOP) were still preserved. Therefore, the distribution pattern of eukaryote-specific intronless genes and the gain and loss patterns in this work are in accord with earlier reports [13, 15, 33]. 

The predicted cellular role of each kind of combination is shown in the supplementary material. Amino acid biosynthesis, cell envelope, energy metabolism, translation, transport and binding are usually well represented. Furthermore, the distribution of basic functional categories, such as amino acid biosynthesis, energy metabolism, and translation, are overrepresented in intronless genes conserved in all major biological kingdoms (ABE) or all eukaryotic groups (FMOP) compared to others.

### 3.4. ORFans

**Figure 6:**
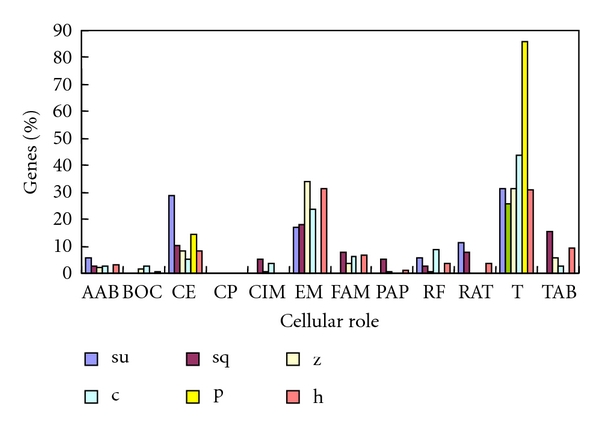
Distribution of ORFans according to their functional categories in each species. The description of functional categories and species is the same as that given for Figure 2.

The protein sequences that have no homologue in other species are termed ORFans [23]. These proteins could be responsible for some species-specific characteristics, and most of these proteins might have evolved faster than others [34]; in fact, they are part of the most interesting genome content. Thus, it is important to experimentally characterize these proteins or use more sensitive bioinformatic approaches to understand their roles and functions [15]. We found very few ORFans in these species except in human (Table 2), about 22.7% of whose intronless genes are ORFans, and this might be due to their complexities because they are viviparous and mammalian. Most of these proteins are annotated as hypothetical; however, majority of ORFans in all species, except platypus, have mRNA or EST supports when we checked their annotations. Thus, most of these ORFans might not be misannotated.  Figure 6 shows the predicted cellular role distribution of ORFans in each species. It is interesting to note that translation and energy metabolism are the most frequently represented cellular roles in these species. The pattern is similar to earlier reports of plants and human [13, 15], suggesting that most species-specific intronless genes in plants and animals have the same functions and even the components of basic cellular machinery might evolve to perform species-specific functions in all these species [13, 15]. Moreover, we found the cellular role of cell envelope is well represented in sea urchin ORFans and more than half of the intronless genes that have the cellular role of fatty acid metabolism are ORFans.

### 3.5. Conserved Intronless Genes in Deuterostomes

**Figure 7:**
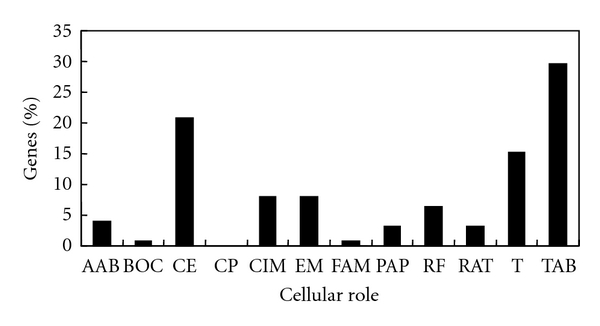
Functional distribution of conserved intronless genes in vertebrates. The description of functional categories is same as that given in Figure 2.

To examine the conservation of intronless genes in deuterostomes, we clustered them together for these 6 species using CD-Hit (identity = 0.3). Only 6 nonredundant sequences (NR) were shared by these species, and they might perform pivotal functions in deuterostomes. The predicted cellular roles were translation for 4 NRs, regulatory function for one NR, and energy metabolism for one NR. The GO categories of these NRs were associated with transcription regulation, growth factor, and transport. When we compared the shared NRs between any two species, we found that sea urchin and sea squirt have less than 100 shared NRs with other species, but the number was more than 200 between any two vertebrate species (data not shown), suggesting that significantly more intronless genes are shared by vertebrates than those shared by deuterostomes. As expected, we found 125 NRs shared by vertebrates, and Figure 7 shows the distribution of the predicted functions of these NRs. Most of these proteins are involved in basic cellular processes, such as transport and binding, cell envelope, and translation, and these genes could be one of the important reasons for the emergence of vertebrates.

### 3.6. Paralogues of Intronless Genes

Intronless genes in eukaryotic genomes have many origins other than inheritance from ancient prokaryotes, such as duplication (whole genome duplication or tandem duplication) of existing intronless genes and retroposition of intron-containing genes (retroduplicated genes). Also, there is evidence that ancient intronless genes were the origin of multiexon genes [35, 36]. To investigate these latter patterns, we clustered intronless genes and nonredundant intronless genes with multiexon genes using CD-Hit (identity = 0.3), and the results were shown in Table 1. It shows that about 92% of sea urchin nonredundant clusters have more than 1 intronless gene and the value is 58% in human, 32% in zebrafish, and only 9% in sea squirt. These data suggest that most intronless genes may originate from other intronless genes in sea urchin, human, and zebrafish, but much fewer intronless genes have the same origin in other species. Table 1 also shows the frequency of correspondence between nonredundant intronless genes and nonredundant intron-containing genes. About 30% of nonredundant intronless gene clusters have corresponding nonredundant intron-containing genes in each species, but in sea squirt and chicken, the proportion is only 15% and 18%, respectively. This might be due to the activity of LINE retrotransposable elements in their genomes. Active LINE retrotransposons that can reversibly transcribe polyadenylated mRNAs are thought to be the main reason for the emergence of retrogenes [37, 38]. More than 20% of the human genome is composed of LINE retrotransposable elements [39], and many studies have suggested a high rate of retroposition in human [26, 40, 41], which might result in the emergence of intronless genes. In chicken, only about 8% of the genome is comprised of the CR1 (chicken repeat 1) [42] and this kind of LINE-1 is not thought to reversibly transcribe polyadenylated mRNAs [42]. Majority of intronless genes that have intronless or intron-containing homologs are associated with cellular roles transport and binding, cell envelope, and translation. It has long been believed that duplicated genes (including retroduplicated genes) provide material for the evolution of genes with new functions [43], but there is evidence that retrogenes function as their parent genes during the spermatogenesis X chromosome inactivation of meiosis in mammals [12] and in the fruit fly [10]. Thus, the selective advantage of retention of these duplicated intronless genes might be that these genes can evolve new functions or help to buffer crucial functions similar to earlier reports on duplicated genes in angiosperms [44].

## 4. Conclusion

Both this and earlier studies indicate that the evolutionary patterns of intronless genes among deuterostomes, as well as between deuterostomes and plants, have many common characteristics and might be appropriate for all major eukaryote kingdoms. However, there are still some lineage-specific and species-specific characteristics on the evolution of intronless genes, and this might be one of the reasons for the existence of biodiversity in this world. As more genome sequences are sequenced and more exhaustive and accurate genes are annotated, the evolutionary patterns of intronless genes will become clearer, providing insights into understanding the evolutionary mechanisms underlying gene or genome evolution in eukaryotes.

## Figures and Tables

**Table 1:** The *C*-values and statistics for genes (intron and intronless) in each species.

Species	Sea-Urchin	Sea-Squirt	Zebrafish	Chicken	Platypus	Human
*C*-value (pg)	0.89	0.20	1.75	1.25	3.06	3.5
*N*	—	19858	40585	22194	26836	88237
*I*	2482 (8.6)	1448 (11.2)	2169 (8.6)	1659 (10.2)	930 (7.9)	6229 (16.7)
NR*	676	1263	856	1029	516	2290
NRI*	8792	8502	10620	9502	7840	12823
R**	621 (92)	110 (9)	274 (32)	156 (15)	86 (17)	1321 (58)
C**	212 (31)	191 (15)	269 (31)	186 (18)	133 (26)	665 (29)

*C*: values are obtained from http://www.genomesize.com/.

*N*: number of proteins encoded by intron and intronless genes, obtained from Ensemble (http://www.ensembl.org/index.html).

*I*: number of intronless genes in each genome, numbers in parentheses are percentages of intronless genes account for total genes.

NR: number of nonredundant intronless gene clusters.

NRI: number of nonredundant intron gene clusters.

*R*: number of nonredundant clusters that represent more than one intronless gene.

*C*: number of clusters that represent both a nonredundant intronless and a nonredundant intron-containing gene.

*Clustered using CD-Hit (identity = 0.3).

**Numbers in parentheses are percentages they account for all nonredundant intronless gene clusters.

**Table 2:** Number of intronless genes with homologous genes in other taxonomic groups.

Taxonomic group	Sea urchin	Sea squirt	Zebrafish	Chicken	Platypus	Human
Archaea	764	625	524	385	143	1184
Bacteria	1303	873	1161	920	345	2524
Fungi	1492	1149	1067	817	413	2513
Plants	1480	1209	1070	809	441	2617
Metazoans	2447	1403	2027	1575	918	4807
Other eukaryotes	2337	1343	1897	1388	867	4055
ORFans	35	39	136	80	7	1416

ORFans [23]: homologues not found in other species.
